# Female fruit flies use social cues to make egg-clustering decisions

**DOI:** 10.1186/s12915-025-02382-w

**Published:** 2025-10-14

**Authors:** Emily R. Churchill, Emily K. Fowler, Lucy A. Friend, Marco Archetti, Douglas W. Yu, Andrew F. G. Bourke, Tracey Chapman, Amanda Bretman

**Affiliations:** 1 School of Biology, Faculty of Biological Sciences, University of Leeds, Leeds, LS2 9JT UK; 2 School of Biological Sciences, University of East Anglia, Norwich Research Park, Norwich, NR4 7TJ UK; 3 Department of Biology, Pennsylvania State University, State College, University Park, PA 16802 USA

**Keywords:** Cooperative oviposition, Laying rate, Public goods, Social density, *Drosophila melanogaster*

## Abstract

**Background:**

The ability to respond plastically to environmental variation is a key determinant of fitness. Females may use cues to strategically place their eggs, for example adjusting the number or location of eggs according to whether other females are present and driving the dynamics of local competition or cooperation. The expression of plasticity in egg-laying patterns within individual patches (i.e. in contact clusters or not) represents an additional, under-researched, and potentially important opportunity for fitness gains. Clustered eggs might benefit from increased protection or defence, and clustering could facilitate cooperative feeding. However, increased clustering is also expected to increase the risk of overexploitation through direct competition. These potential benefits and costs likely covary with the number of individuals present; hence, egg-clustering behaviour within resource patches should be socially responsive. We investigate this new topic using the fruit fly *Drosophila melanogaster*.

**Results:**

Our mathematical model, parameterised by data, verified that females cluster their eggs non-randomly and increase clustering as group size increases. We also showed that as the density of adult females increased, females laid more eggs, laid them faster, and laid more eggs in clusters. Females also preferred to place eggs within existing clusters. Most egg clusters were of mixed maternity.

**Conclusions:**

Collectively, the results reveal that females express plasticity in egg clustering according to social environment cues and prefer to lay in clusters of mixed maternity, despite the potential for increased competition. These findings are consistent with egg-clustering plasticity being selected due to cooperative benefits.

**Supplementary Information:**

The online version contains supplementary material available at 10.1186/s12915-025-02382-w.

## Background

In natural contexts, social environments and availability of resources are highly variable, exposing populations to fluctuating competition. In response, phenotypic plasticity can play a key role in ensuring individuals maximise their lifetime reproductive success [1, 2]. Because optimal strategies may vary with fluctuating levels of competition, individuals who respond to the prevailing environment should show increased fitness. Plasticity in investment decisions should be particularly important for females, who can generally exert greater control over reproductive investment in progeny [3–8]. For example, for females that deposit eggs upon a substrate, choice of location can be a vital determinant of hatching success. Consistent with this, female aggregation and oviposition behaviours of many species appear to be plastic in response to environmental variation [9–12].

### Plasticity in aggregation and egg laying — competition, cooperation, and cheating among females

Oviposition choices by females may influence the opportunities for competition and cooperation between offspring. When food is limited and competition is high, eggs and larvae are vulnerable to cannibalism, whereas larvae can benefit from increased efficiencies resulting from communal feeding [13, 14]. Females can use the presence of conspecifics as a cue to modify reproductive investment in response to changing social conditions. For example, in the willow leaf beetle (*Plagiodera versicolora*), increasing adult female density results in a decrease in egg hatching but an increase in egg clutch number and duration of laying period [15].

Females that deposit their eggs upon substrates can often lay eggs in contact with each other, i.e. in clusters, e.g. in fish [16], birds [17], reptiles, amphibians [18], and invertebrates [19], which may further exacerbate competition or cooperation. Benefits from cooperation could include reduced energy expenditure for ovipositing females by minimising search times, reduced egg predation risk (the dilution effect hypothesis [20]), increased protection from abiotic challenges, increased larval communal feeding, or protection through antipredator or antimicrobial defensive compounds [21–23].

These defensive substances represent potential public goods [24, 25] as all nearby eggs could benefit, including those not provisioned with defensive compounds. In any public goods system, cheaters (individuals hoping to gain shared benefits without the costs of contributing to the resource) and cooperators could stably coexist within a population if the benefits of public goods have a non-linear relationship with fitness [25, 26]. This raises the intriguing possibility that in egg clusters of mixed maternity, there could be cooperator eggs coated with defensive compounds and undefended cheater eggs that benefit from placement within the defensive diffusion radius.

The potential fitness benefits of joining an existing egg cluster within a food patch could also depend on key factors such as number, quality, fertility, and age of eggs already present. Based on existing evidence that females can vary the number of eggs they lay according to social density [9, 10, 27], we predict and test here in the *Drosophila melanogaster* fruit fly model system the idea that females use the number of eggs and/or number of adult conspecifics as cues to direct their egg-clustering patterns.

### *Drosophila melanogaster* as a model for understanding plasticity in oviposition

In wild populations, *D. melanogaster* larvae and adults periodically occur at high densities around fallen, fermenting fruit. For females, fruit is both a source of nutrition and a potential oviposition site. In *Drosophila* and other species in which fruit resources are patchy, ephemeral, and vary in nutrient availability (density, ripeness, and state of decomposition), access to oviposition sites is likely to be shaped by the immediate social environment (number of conspecific or heterospecific males and females utilising the same resource) [28]. To oviposit in a way that maximises fitness, females should assess and respond appropriately to the nutritional quality, degree of interspecific competition, risk of pathogens and parasites present at each oviposition site, and the ongoing search costs and potential benefits and costs for developing offspring [21].

Consistent with this reasoning, previous research shows that female *Drosophila* can show attraction to conspecifics that are utilising specific oviposition resources [22, 23], which they can detect directly or via pheromones, markings, or the presence of eggs or larvae [29–33]. Several studies show that gravid females can aggregate to lay their eggs *between* substrate patches used by others according to a variety of physical and social environmental conditions [9, 34–36]. This attraction could arise because it allows females to copy the site-selection choices of others [29, 30, 32] to enable cooperative feeding among larvae [37] or to gain other potential public goods benefits. Female *Drosophila* can adjust the number of eggs they lay according to their previous or current social environments, laying fewer eggs after exposure to conspecifics before mating [9, 38] but laying eggs more quickly if they are in larger social groups after mating [27].

**Fig. 1:**
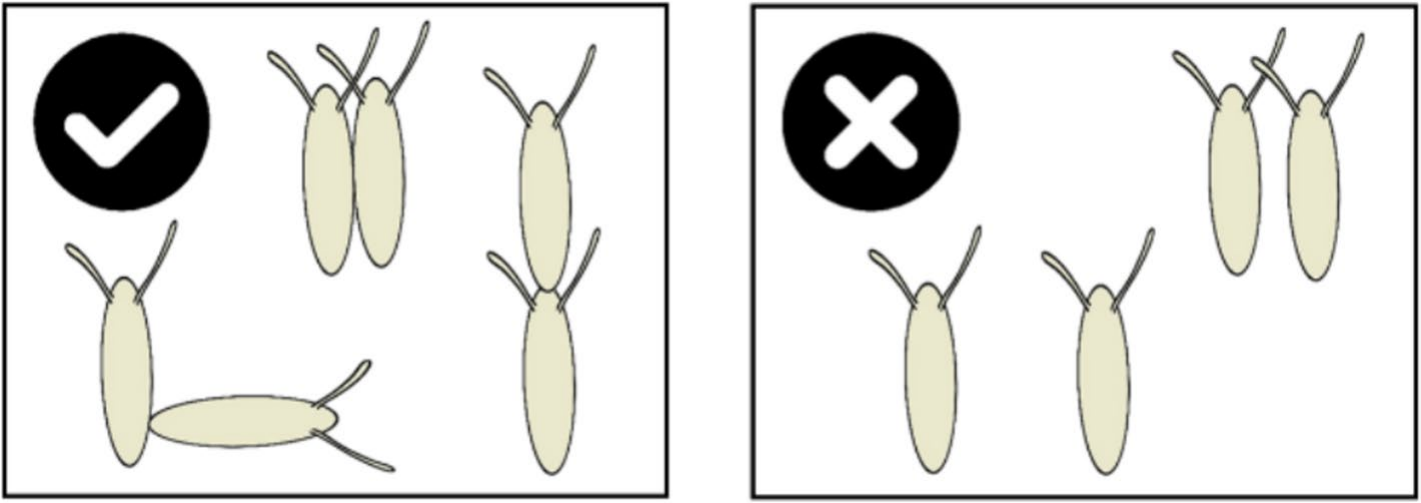
Egg clustering is defined as eggs in direct contact. Eggs where the main bodies were in direct contact (left) were classified as clustered, and those not in contact (or where respiratory appendages only overlap) were classified as singly laid (right)

*Drosophila* eggs can often be found in extremely close proximity, in large clusters (Additional file 1: Fig. S1). Hence, after deciding to lay on substrates with existing conspecific eggs, females must also decide whether to lay eggs that join existing clusters. Females generally lay one egg at a time [39], explore oviposition sites between laying, and can retain eggs until an optimal laying site is found [40]. Therefore, the clustering of eggs suggests that females make repeated egg-laying decisions, and that females lay their eggs in nonrandom distributions. It is unknown whether the temporal or spatial distribution of egg laying is significantly non-random, how egg-laying location choices vary *within* a patch, or whether the factors influencing female egg-laying patterns differ *within* patches. The pattern of egg laying within patches can be examined by assessing egg-clustering patterns, which we define as two or more eggs in direct contact (Fig. 1). Egg clustering is expected to be important, as it offers females the opportunity to optimise fitness by placing their eggs in a manner that maximises benefits of cooperation and the potential for public goods benefits (e.g. protection, defence, or cooperative tunnelling) against potential costs of competition (e.g. overexploitation of food). Clustering by our definition also potentially enables further benefits only transferred via eggs being in direct contact.

We tested these ideas by addressing the following three hypotheses (H):

1. That female *D. melanogaster* lay their eggs non-randomly within a food patch in relation to other eggs.
2. That changes to adult social density, and the presence of existing egg clusters, alter egg-clustering patterns by individual females.
3. That females lay eggs in clusters of mixed maternity at a high frequency, cooperating with other gravid females present.

Whilst studies have shown aggregation *between* patches previously [9, 42–44], whether egg-laying patterns *within* patches are non-random has not previously been explored. Therefore, we first constructed a mathematical model, parameterised by experimental data, to test the hypothesis that females exhibit nonrandom egg placement (H1). We then conducted additional experiments to determine key drivers of egg-laying patterns observed. We examined whether egg-clustering patterns responded plastically to adult female group size or to the density of eggs already present in the local environment (H2). Potential advantages of site copying and egg clustering [9, 13, 14, 20, 27, 30, 37, 41–45] could lead to females increasing egg clustering in response to higher densities of adult females and already-laid eggs in the environment. Females could potentially maximise public goods-related benefits under increased clustering by laying eggs alongside those of others. To establish whether conditions for this scenario exist, we examined whether females do indeed cluster eggs within patches with those of conspecifics by using dyed eggs to distinguish eggs laid by different females (H3).

## Results

### Egg-laying patterns were significantly non-random, and clustering increased with increasing group size (H1 and H2)

Using our model, we explored whether oviposition distributions were significantly non-random, and how clustering preferences varied with increasing group size, to understand whether egg-laying location decisions were plastic. Across the four social treatments, clustering preferences (values of К that, based on the Kolmogorov–Smirnov test, yielded no significant difference between the simulated and observed distributions) were between 0.3 and 0.5, and clustering preferences increased with increasing female group size (Additional file 1: Table S2).

**Fig. 2:**
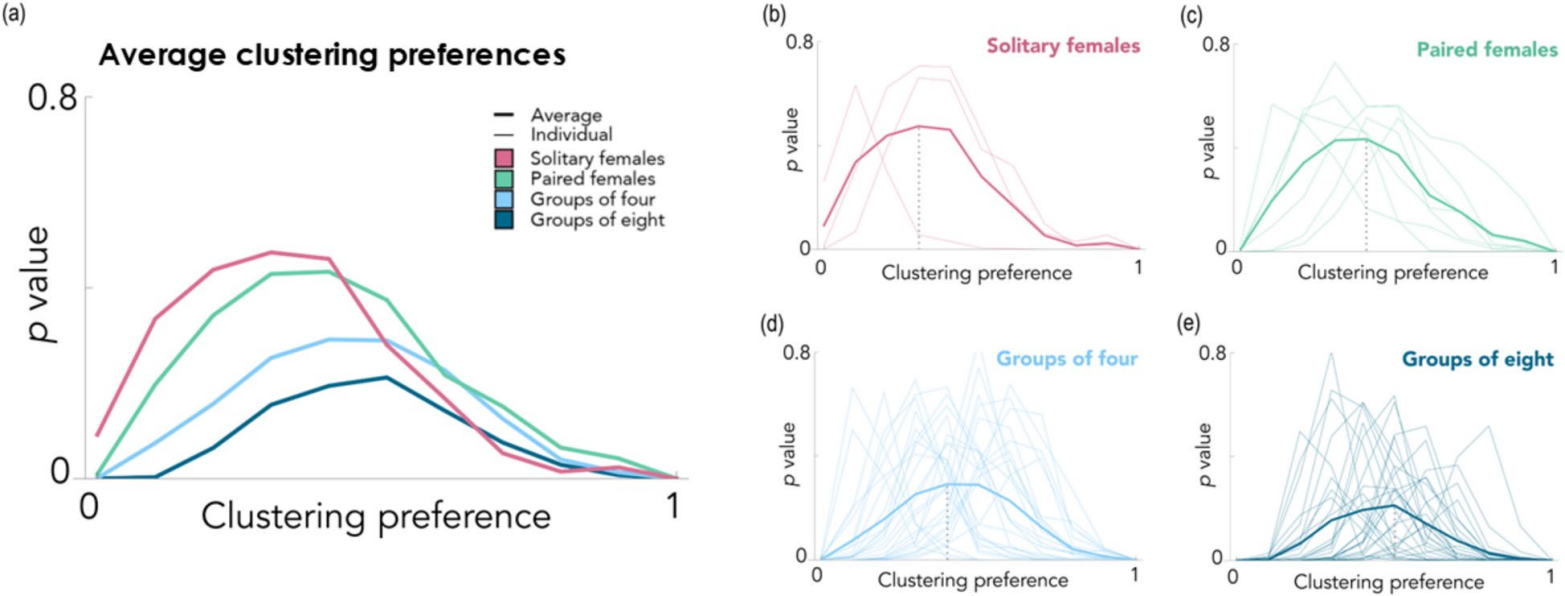
Egg clustering is non-random and increases with increasing adult social density. **a** Comparison of the clustering proportion model outputs and empirical egg laying patterns observed in *D. melanogaster* for four social group sizes (one, two, four, and eight adult females per group). The average Kolmogorov–Smirnov *P*-values at К (clustering preference) values ranging from 0 to 1 in 0.1 increments — where the null distribution is *К* = 0 (no eggs clustered) and *К* = 1 (all eggs laid in one large cluster). The best-fit values of *К* are those that produced the least significant (highest P value) difference between simulated and observed data—i.e. the peak of each curve. **b**, **c**, **d**, **e** The Kolmogorov–Smirnov *P*-values for individual replicates in the four social group sizes (one, two, four, and eight adult females), with the values for each vial shown in the pale, thin lines and the average value indicated with a bold line. The vertical grey dotted lines show clustering preference values

For all social treatments with two or more females, patterns of egg clustering in the empirical data were significantly more clustered than those predicted by the null model (H1; Wilcoxon signed-rank test comparisons with simulations using *К* = 0; paired: *z*_(6)_ = − 2.29, *p* = 0.0220, *r* = 0.866, *N* = 7 vials; groups of four: *z*_(25)_ = − 4.47, *p* = 7.95 × 10^−6^, *r* = 0.876, *N* = 26 vials; groups of eight: *z*_(27)_ = 4.63, *p* = 3.63 × 10^−6^, *r* = 0.875, *N* = 28 vials). Therefore, egg-clustering patterns of all grouped females followed a nonrandom distribution (Additional file 1: Table S2, Fig. 2a, b, c, d, e), but those of solitary females did not (*z*_(2)_ = − 1.36, *p* = 0.174, *r* = 0.786, *N* = 3 vials), suggesting that their laying patterns were random. Although we note that parameters were more difficult to estimate for the solitary social treatment as clustering was observed only infrequently under those conditions, only 3 out of the 30 vials could be included in this analysis due to the low clustering rates observed by isolated females. The findings support H1, indicating that eggs were laid in nonrandom patterns, which became more non-random as social density increased. Therefore, egg-clustering patterns were plastic and responsive to the female’s social environment.

### Egg-clustering patterns responded both to the density of adult females and the presence of already-laid eggs in the environment (H2)

#### (i) Females in larger social groups clustered their eggs more and laid more eggs at a faster rate

##### Social group size and likelihood to lay eggs

Using the empirical data from the final timepoint (to allow all females maximum laying opportunities) shown in Fig. 2a, b, c, d, and e, we found that females in larger social group sizes were significantly more likely to lay eggs (generalised linear model (GLM): *χ*^2^ = 20.2, *df* = 116, *p* = 1.51 × 10^−4^). Eggs were present in 100% of vials housing eight females, a significantly higher proportion than for vials containing a solitary female (66.7%, GLM: *χ*^2^ = 15.9, *df* = 58, *p* = 6.76 × 10^−5^) or those with two females (83.3%, GLM: *χ*^2^ = 7.39, *df* = 58, *p* = 0.00657). There were also significantly more vials containing eggs from the four-female treatment (96.7%) compared to the solitary female treatment (GLM: *χ*^2^ = 10.2, *df* = 58, *p* = 0.00140).

##### Social group size and number of eggs laid

**Fig. 3:**
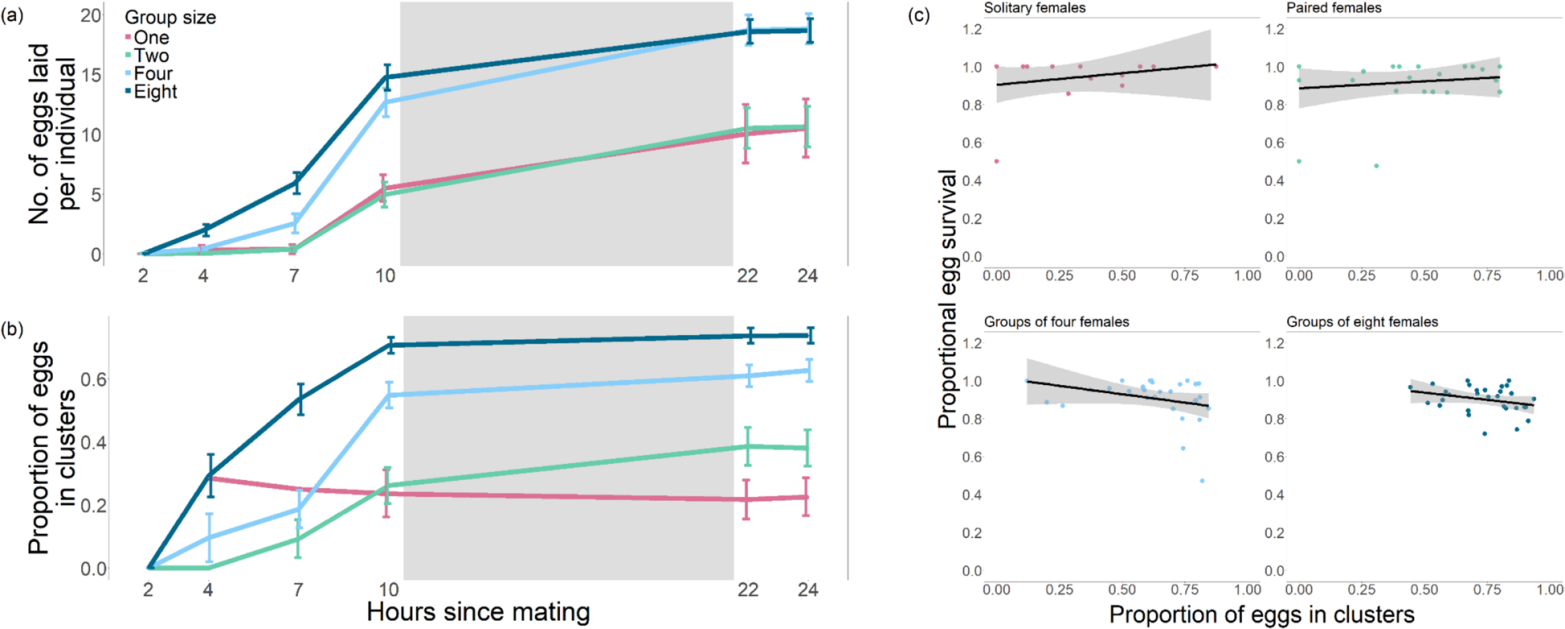
Females in larger groups laid more eggs, more quickly, with a higher proportion of clusters. **a** Females housed in groups of four and eight were quicker to lay and laid more eggs per female than those kept in solitude or in pairs (GLM: all *N* = 30 vials). Eggs laid per individual were calculated by dividing the total number of observed eggs by the number of females in the vial. Social treatment means and standard errors are shown for the four housing densities at each observation time point. The grey box indicates the period of dark (21:00–09:00 GMT). **b** Females housed in groups of four or eight laid a higher proportion of their eggs in clusters compared to those kept in solitude or in pairs (LM: all *N* = 30 vials). Proportion of eggs clustered was calculated by summing all cluster sizes and dividing by the total number of eggs counted. **c** There was no effect of the proportion of egg clustering on egg-adult viability (linear regressions with 95% confidence intervals; solitary: *N* = 20 vials; paired: *N* = 24 vials; groups of 4: *N* = 29 vials; groups of 8: *N* = 30 vials)

Females in larger social group sizes also laid more eggs per female than did the females from the other treatments (solitary females: 7 ± 10 eggs per female (*N* = 30 vials); pairs: 9 ± 9 eggs per female (*N* = 30 vials); groups of four: 18 ± 8 eggs per female (*N* = 30 vials); groups of eight: 19 ± 5 eggs per female (*N* = 30 vials); GLM: *F*_3, 116_ = 265, *p* = 2.93 × 10^−8^; Fig. 3a). Consistent with this, post hoc tests showed that females in solitary and paired social densities laid fewer eggs than females in groups of four and eight (GLM: all *p* < < 0.001). To control for the increased sampling effort in treatments with larger groups of females, the analysis was repeated with a randomised subset of data to make the number of flies (rather than the number of vials) in each treatment similar. The results of these analyses were comparable with the main analysis (larger social group sizes showed a significantly higher likelihood of egg laying: GLM: *χ*^2^ = 50.0, *df* = 54, *p* = 0.0271 and significantly higher numbers of eggs laid: GLM: *F*_3, 54_ = 137, *p* = 0.00427).

##### Social group size and proportion in clusters

An analysis of the data at the final timepoint (24-h post-mating) supported the clustering analysis described in the previous section. At this timepoint, the first eggs laid after mating would begin to hatch, altering potential benefits to clustering and thus likely changing location decisions. Plus, once eggs began to hatch, they would disrupt existing clusters, so it was not possible to collect accurate location decision data after this point. Females housed in larger groups laid a higher proportion of their eggs in clusters (solitary females: 22.6% ± 26.9% (*N* = 20 vials); pairs: 38.1% ± 28.9% (*N* = 25 vials); groups of four: 62.7% ± 19.2% (*N* = 29 vials); groups of eight: 73.7% ± 13.3% (*N* = 30 vials); total number of eggs laid included in the model: linear model (LM): *F*_3, 99_ = 28.4, *p* = 2.53 × 10^−13^; all pairwise comparisons *p* < 0.05; Fig. 3b). We included the total number of eggs laid in the model as we found that there was a positive relationship between the number of eggs and the proportion of eggs clustered (LM: *F*_1, 99_ = 6.66, *p* = 0.0113). This result is expected because as the number of eggs increases, so does the potential for egg clustering, although this was not the only driver of social density effects on clustering proportions as the statistical results indicate.

##### Social group size and proportion in clusters over time

In all grouped female treatments, the proportion of clustering also increased over time (individual vial included in the model as a random effect; linear mixed-effects models (LMEs): pairs: *F*_1, 58_ = 19.1, *p* = 5.26 × 10^−5^; groups of four: *F*_1, 95_ = 75.8, *p* = 9.99 × 10^−14^; groups of eight: *F*_1, 111_ = 74.7, *p* = 4.45 × 10^−14^). There was no significant effect of time on the proportion of clusters in eggs laid by solitary females (LME: *F*_1, 40_ = 1.15, *p* = 0.289). Social group sizes of two and four females displayed less clustering than solitary females in the first 10-h post-mating; however, total egg numbers were low here due to slow laying rates, and so potential opportunities for clustering may have been limited. Further research is needed to clarify whether this laying pattern is robust.

##### Social group size and number and size of clusters

Consistent with this, the number of clusters and size of the largest cluster increased with both increasing social density (LMs: number: *F*_3, 376_ = 463, *p* = 2.20 × 10^16^; size: *F*_3, 299_ = 58.3, *p* = 2.20 × 10^−16^; Figs. S3 and 4) and time (number: *F*_1, 376_ = 631, *p* = 2.20 × 10^−16^; size: *F*_1, 299_ = 67.1, *p* = 7.62 × 10^−15^). These results were robust to differences in sampling effort as shown by an additional analysis using the same number of individuals (rather than vials) at the final timepoint. The effects remained: females in larger groups laid a higher proportion of their eggs in clusters (LME: *F*_3, 41_ = 6.40, *p* = 0.00117), and the number and size of clusters still increased with increasing social density (LMs: number: *F*_3, 134_ = 199, *p* = 2.20 × 10^16^; size: *F*_3, 102_ = 25.1, *p* = 2.96 × 10^−12^).

##### Clustering and fitness effects

Interestingly, there was no evidence for a fitness benefit for clustered eggs in terms of increased egg-adult survival (Fig. 3c). Here, a positive relationship would show that clustering eggs increased female fitness, and a negative relationship that it decreased fitness. There was no effect of social density (one, two, four, or eight females) on the proportion of eggs that survived to adulthood (GLM: *F*_3, 99_ = 0.292, *p* = 0.831; Additional file 1: Fig. S5), and there was no correlation between the proportion of eggs laid in clusters and egg-adult offspring viability (*t* = − 0.485, *df* = 101, *p* = 0.629; Fig. 3c).

Overall, the results supported the hypothesis that the egg-clustering behaviour of females within a food patch responds plastically to the social environment, with females laying eggs more quickly, laying more eggs, and laying more eggs in clusters, as the social group size increases. However, there was no indication that eggs laid in clusters had higher egg-adult viability.

#### (ii) Females preferred to lay eggs within existing clusters rather than with singly laid eggs

Given that females respond plastically to adult social density, we predicted that the density of existing eggs in the laying environment would also provide crucial social information that altered female egg-laying location decisions. As expected, female egg-clustering behaviour showed plasticity in response to eggs already present in the environment. For clarity, here we refer to the manipulated treatment eggs present on the substrate before the addition of female flies as existing eggs/clusters and eggs laid by focal females as ‘new eggs’.

##### Existing egg clusters and presence of new egg clusters

**Fig. 4:**
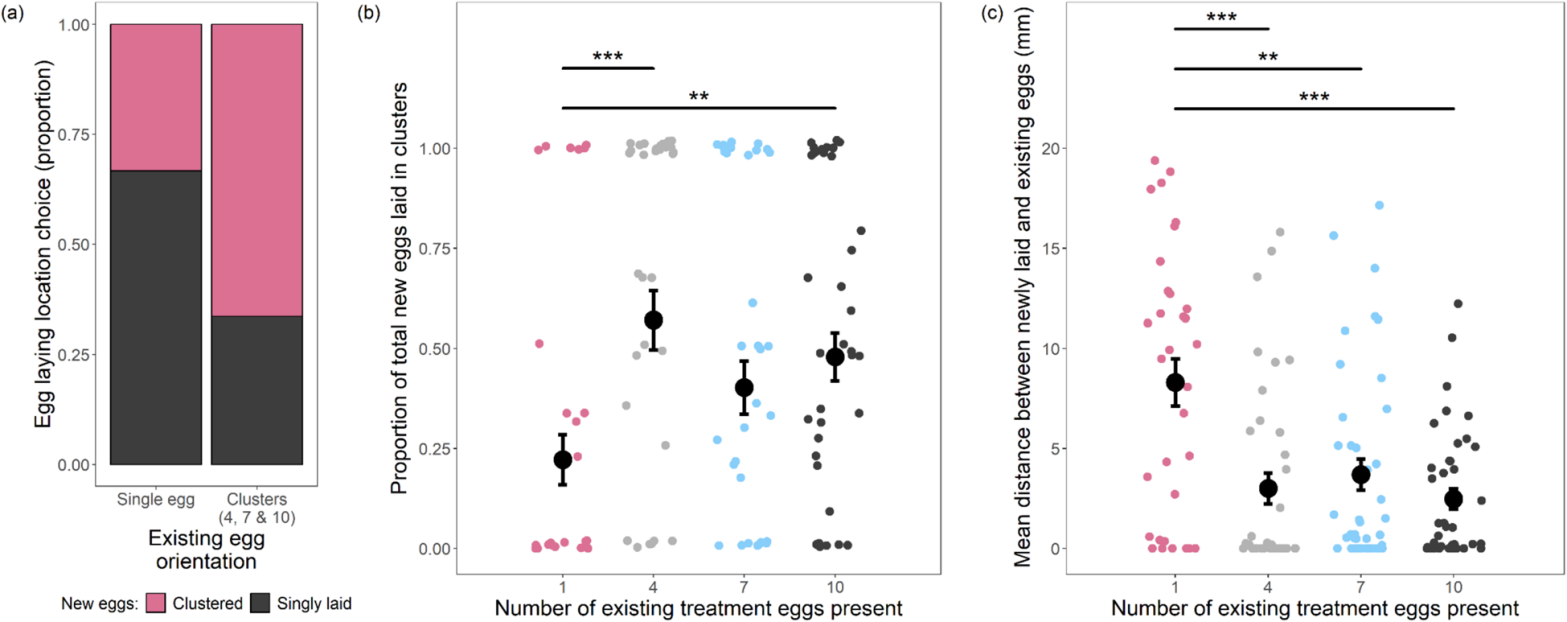
Females lay more eggs within existing clusters and lay eggs closer to existing egg clusters. **a** A higher proportion of females clustered at least one of their eggs with existing eggs when those existing eggs were in clusters compared to singly laid eggs (singly laid eggs: *N* = 36; clustered eggs: *N* = 122). **b** Females laid a higher proportion of their eggs in clusters with existing eggs when those existing eggs were already in a cluster (of any size) compared to a singly laid egg (1 egg: *N* = 36; 4 eggs: *N* = 37; 7 eggs: *N* = 41; 10 eggs: *N* = 44). Means and standard errors are shown for the four existing treatment egg cluster sizes. Significant differences between existing treatment egg cluster sizes are represented by an overarching bar; * indicates a significant difference between paired treatments (LMs: *P < 0.05, **P < 0.01, ***P < 0.001). **c** Mean distance of laid eggs from existing treatment eggs was greater when the number of existing eggs was one compared to egg clusters of any size (1 egg: *N* = 32; 4 eggs: *N* = 37; 7 eggs: *N* = 40; 10 eggs: *N* = 40)

Females were more likely to add at least one of their eggs to existing clusters of 4, 7, or 10 eggs than they were to lay with a singly placed existing egg (GLM: *χ*^2^ = 12.5, *df* = 156, *p* = 4.18 × 10^−4^; Fig. 4a). The total proportion of eggs laid that joined existing eggs were also higher when existing eggs were clustered (LM: *F*_3, 154_ = 4.75, *p* = 0.00340; Fig. 4b). Proportions were calculated from the number of eggs females were able to lay within the 30-min observation window (between 1 and 19 eggs per vial). Post hoc tests showed that egg cluster sizes of 4 and 10 were joined significantly more often than were single eggs (LMs: 4: *F*_1, 71_ = 13.0, *p* = 5.85 × 10^−4^; 10: *F*_1, 78_ = 8.76, *p* = 0.00407), though this trend was marginally non-significant for a cluster size of 7 (LM: *F*_1, 75_ = 3.82, *p* = 0.0542).

##### Existing egg clusters and distance of new eggs to existing eggs

In addition to scoring whether a laid egg was physically in contact with an existing egg (i.e. clustered), we also measured the distance between each newly laid egg and existing treatment eggs already present on the substrate. We did this to test for potential benefits of close proximity in addition to direct contact. Females laid their eggs closer to existing clusters (H3; LM: *F*_3, 145_ = 9.86, *p* = 5.86 × 10^−6^; Fig. 4c) with all three cluster sizes having shorter inter-egg distances than eggs from females exposed to single eggs (LMs: 4: *F*_1, 67_ = 14.8, *p* = 2.68 × 10^−4^; 7: *F*_1, 70_ = 11.4, *p* = 0.00122; 10: F_1, 70_ = 23.8, *p* = 6.57 × 10^−6^). Hence, laid eggs that did not strictly join a cluster were still laid closer to existing clusters than to single eggs. The results show that females also respond plastically to the clustering patterns of eggs already present in the environment and, consistent with the results above, lay their eggs preferentially within existing egg clusters (H3).

### Egg clusters were typically of mixed maternity (H3)

**Fig. 5:**
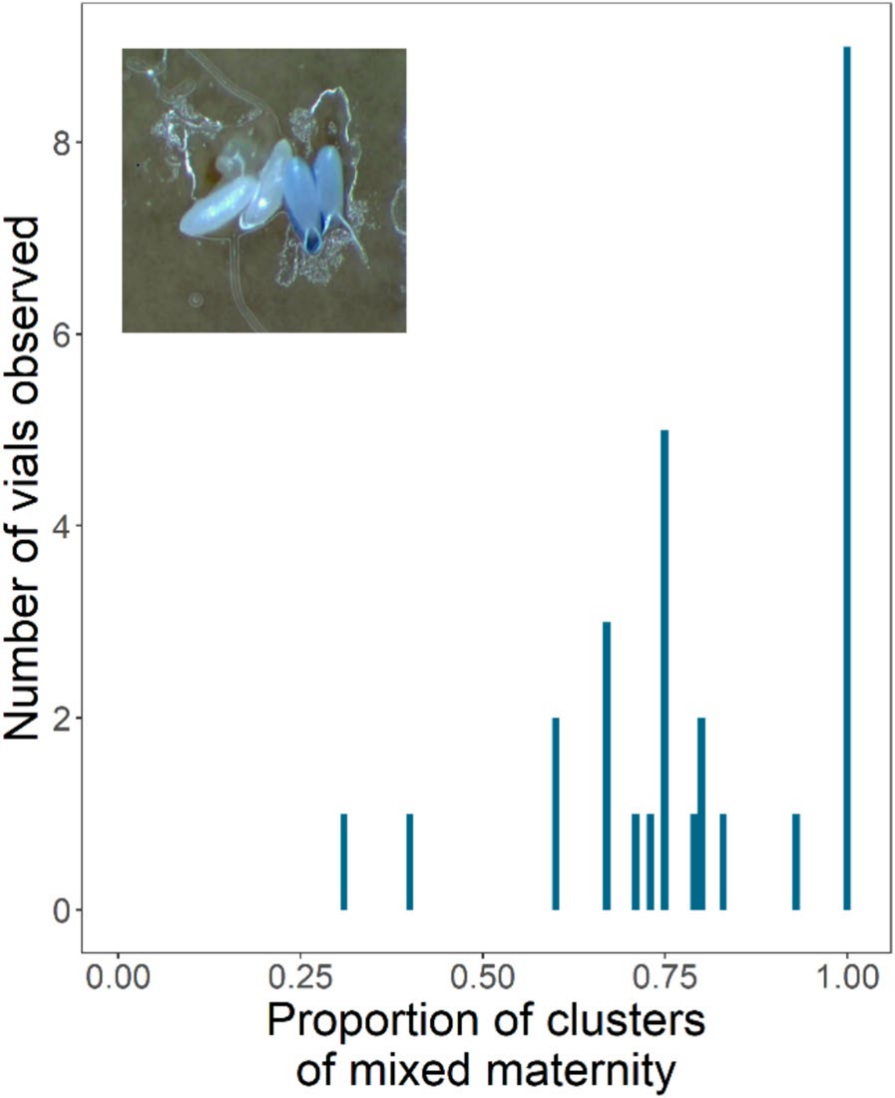
High frequency of mixed maternity egg clusters. Females showed no preference for laying their eggs in single-maternity clusters, as 79.5% of egg clusters observed were of mixed maternity (*N* = 28 vials). Shown is a frequency distribution of the proportion of egg clusters counted that had eggs laid by two or more gravid females. The inset picture shows a cluster of four eggs: two undyed and two dyed with Sudan Black B dye

Having previously demonstrated that females prefer clusters over singly laid eggs, in our final experiment, we wanted to better understand the potential benefits of clustering by exploring whether clusters tended to be of single or mixed maternity. To do this, we used a lipophilic dye to enable us to distinguish between eggs laid by different females [46]. We set up groups of four females comprising one standard-fed focal wild type and three Sudan Black B dye-fed non-focals. We found that of the focal eggs that had been laid in clusters, a mean of 79.5% of them were in mixed-maternity clusters (Fig. 5). This showed that females do not preferentially choose to isolate their eggs by laying them solely in single-maternity clusters but instead lay eggs in clusters of mixed maternity at high frequency.

## Discussion

The main new findings of this study were that female egg-clustering behaviour within patches responded plastically to the social environment and the presence of existing eggs within the oviposition environment. All three hypotheses were supported.

1. Egg-clustering patterns were significantly non-random for females in groups, but not for eggs laid by socially isolated females.
2. As the social group size of females increased, females laid eggs more quickly, laid more eggs, and laid more of them in clusters. However, there was no evidence of a fitness benefit to clustered eggs in increased egg-adult viability.
3. Clusters of eggs were usually of mixed maternity, and females preferred to add their eggs to existing clusters. Overall, females showed striking plasticity in their egg-clustering decisions, with a strong bias towards laying eggs in mixed-maternity clusters. However, the fitness benefits of such behaviour remain elusive. These results are explored in more detail below.

### Egg-clustering decisions are plastic and eggs are laid in non-random patterns

For all females except those held in social isolation, egg clustering occurred more than the chance outcomes predicted by the model. This provides evidence that females make active egg location decisions, and that these responses are plastic and change in response to varying social environments. Egg clustering shows that females make repeated egg-laying decisions, as they generally explore potential laying sites between laying each individual egg and can retain their eggs until they find an optimal site [39, 40]. Exactly how female *Drosophila* detect existing clusters of eggs has yet to be discovered. However, there is evidence that *Drosophila* use chemical cues to detect egg and larvae presence [30, 33, 47] and olfaction to detect surrounding microbes [46, 48] when assessing general laying sites, but how this strategy compares to detecting local-scale clustering is unknown. Understanding how females detect these clusters could help to identify the benefits of these behaviours.

Increased clustering has the potential to increase local population density within patches, which has potential cumulative effects, as even relatively small-scale variation in density leading to increased competition can have consequences for fitness [49]. For this reason, it could be expected that females would take the opposite approach: those in larger social group sizes could retain eggs until they found a suitable oviposition site without competitors. However, this is not the case, as laying rate increased in larger social group sizes. This could be because test vials were at a low density compared to population cultures and were not overcrowded. The vials in theory had the capacity to hold 5770 eggs, but the maximum number of eggs laid was 233 so all vials had space remaining to cluster. A previous study showed no density effects on body size or reproductive traits when up to 1000 larvae were reared on the same housing conditions [50].

It is possible that clustering will occur more frequently at the edge of a laying resource given their established preference for this space [9, 44, 45]. However, it is not yet known whether females held under the social conditions used here show edge preferences for egg laying; hence, it was not possible to include this in our model. Ongoing research into edge effects is required to fully explore how these interact with social effects.

### Females cluster more of their eggs in larger groups and more commonly form mixed-maternity clusters

The proportion of eggs that were laid in clusters increased as social density increased. Although there was a higher chance of clustering when there were more eggs present in the environment (also see [46]), this increase in clustering with increasing social density did not depend upon egg number (in agreement with the null model comparison).

It was not possible to determine the maternity of the clusters in grouped treatments in the initial experiment, but our later experiment showed that the clusters are usually of mixed maternity. Females exposed to other (non-kin) females preferred to add their eggs to mixed-maternity clusters, and overall socially isolated females cluster their eggs much less, which suggests that there are potential benefits of laying in mixed-maternity clusters over and above laying in clusters per se. The drivers of this are not yet known; however, females could be balancing a bet-hedging strategy of laying in multiple sites to reduce risks of sibling competition (if food is limited) or to minimise predation/parasitism risk by reducing transmission and egg visibility [51] whilst adding to existing clusters to reap potential public goods benefits of acquiring more diverse microbiomes [52, 53] or diffusible defensive compounds such as anticannibalism pheromones [43] or antimicrobials (as seen in medfly [41]) (see [46]). Mixed-maternity egg clustering could select for cheating among females under a public goods system [24, 25], a possibility that would be interesting to test further. In this scenario, cheaters might benefit by avoiding the energetic expense of provisioning eggs with defensive compounds. Egg clustering might also provide benefits as well as cheating opportunities through communal feeding. For example, if eggs are laid on hard substrates, there could be benefits for all via the collective processing of food whilst simultaneously allowing the possibility for cheaters to benefit by utilising resources liberated by the processing of food by their cluster mates.

Females in pairs and group sizes of four seemed to have a lower clustering preference in the first 10-h post-mating. If this is a real phenomenon, this could be evidence that females experience an adjustment period in changing social environments, and so clustering responses are slower in groups. This effect could potentially be overcome in increasingly larger groups — e.g. in the social group size of eight females. A more detailed investigation into laying rates and patterns would be required to fully understand this.

### Females preferred to lay their eggs in existing egg clusters

Females preferred to pile their eggs or lay them close to existing clusters of any size. Interestingly, the strength of this preference did not increase with increasing cluster size. This could be because (i) there is no benefit to increasing cluster size (i.e. benefits to joining clusters are not additive), (ii) the clusters here did not vary sufficiently in size, or (iii) there is some trade-off to the benefits of clusters of these sizes. For example, eggs laid later may be at a higher risk of cannibalisation [43, 54], and the risks and costs might increase with cluster size. Costs of laying in mixed maternity clusters are also possible if being surrounded by kin provides benefits via communal feeding of larvae [13]. Exactly how individuals might identify maternity of egg clusters within patches, whether by detecting conspecific pheromones [33, 55, 56]) or absolute cluster size, remains to be investigated. The strong aversion to lying next to isolated eggs raises the question of how clusters are initiated in the first place. The females’ choice to copy and lay new eggs by previously laid eggs could require a threshold of a repeated decision (i.e. at least two eggs at the same site) because this is a more robust indicator of site quality than the presence of a single egg. Females may avoid laying with existing single eggs if those already present have a higher probability of infertility, thus offering potentially fewer benefits. However, we found no relationship between cluster size and egg-to-adult viability, which argues against this idea. In addition, whether females can detect infertile eggs is not known, although those laid by virgins have a distinct pheromonal profile [33]. One scenario is that clusters form if the first two eggs are laid by the same mother. Arguing against this idea is that clusters of two eggs laid by different mothers were observed in our mixed-maternity investigation. This suggests that further studies on clustering initiation are required.

### Potential fitness benefits of egg clustering remain elusive

Interestingly, we detected no increase in egg-adult viability with increasing egg clustering. It is not clear why we did not observe fitness benefits, given the potential costs of both maintaining plasticity [57, 58] and increasing local competition [21, 43, 49, 51–54, 59]. It is possible that as mothers were able to choose a clustering strategy, all individuals chose the optimal strategy. To test this, future work could force mothers into making an incorrect choice via a mismatch of environments. It is also possible that the ad libitum food provided obscured any costs of choosing the ‘wrong’ egg-laying strategy. Females could lay many eggs even when resources are scarce (adopting a raffle theory approach to ensure sufficient offspring survive). However, given that the vials in our experiments contained ad libitum food, resource limitation is unlikely to be the cause of any responses observed here. It was necessary to remove females from the egg-laying environments before they had oviposited all fertile eggs, as eggs needed to be counted prior to larvae hatching, which takes place after 22–24 h at 25 °C [60, 61]. Thus, it is also possible that differences in egg-adult viability could have been detected if females were able to continue laying if the benefit only occurs in clusters of mixed-age eggs.

### Egg number and laying rate also increased as adult female density increased

As expected, based on existing research [27], females in larger social group sizes laid more eggs and laid them more quickly. This is consistent with raffle theory, in which the production of more eggs when competition is high ensures that mothers have a greater chance of at least some of their offspring surviving to adulthood [62]. With a greater number of conspecifics present in the environment, there should also be a higher chance of copying behaviours arising, because a greater number of potential ‘initiators’ are present [29, 30, 32]. Increased copying could increase laying rates, benefitting mothers as it may reduce sampling times needed to locate optimal oviposition sites. Consistent with this, we observed that latency to lay the first egg was also shorter at higher adult densities. In situations in which larval density is high and resources are limiting, later-laid eggs could have reduced survival due to resource degradation and cannibalisation [27, 54, 59]. Thus, in high-density environments, mothers should ensure they begin laying quickly to avoid increased offspring lethality.

Previous research [27] showed that the daylight period can inhibit egg laying in *Drosophila*, an effect that can be overridden by being in a group (five females). Intriguingly, our results showed remarkably similar laying rates for isolated and paired females (whereas groups of four or eight females matched laying times reported in [27]), suggesting that exposure to only one other gravid female does not counteract the previously described light-induced inhibition of egg production. This is surprising given that in male *Drosophila*, exposure to one other rival is enough to facilitate reproductive behavioural responses [63–65]. This may suggest that there are sex differences in responses to local population density.

## Conclusions

Overall, we demonstrated that gravid *D. melanogaster* females express plasticity in egg-clustering decisions, laying eggs in a significantly nonrandom manner and responding to differences in social density. Exposure to higher adult social density led females to lay more eggs, at a faster rate — and lay a greater proportion of their eggs in clusters. Exposure to existing egg clusters also led to a higher frequency of clustering decisions, regardless of existing cluster size. We hypothesise that eggs may be clustered to gain public goods benefits. However, the overall ecological importance of this plasticity remains unknown. These results demonstrate that egg-laying and -clustering decisions are highly sophisticated and add to the growing evidence that even non-social organisms such as *D. melanogaster* have unexpectedly rich social lives [66, 67].

## Methods

### Fly husbandry and rearing of experimental flies

Fly rearing and all experiments were conducted at 25 °C on a 12-h light–dark cycle (09:00–21:00 GMT). Wild-type flies originating from a large Dahomey laboratory population maintained in cages with overlapping generations were used throughout. We collected eggs from cages using Petri dishes (*Sarstedt no. 82.1473.001*) filled with grape juice agar-based medium (50-g agar (*Fisher Scientific no. 10048991*), 600-ml red grape juice (*Young’s Brew red wine enhancer*), and 42-ml Nipagin solution (methylparaben, 10% w/v solution, dissolved in 95% ethanol) per 1.1 l H_2_O). Once eggs had hatched, we transferred first instar larvae to a 40-ml plastic vial (*Sarstedt no. 58.490*) containing 7 ml of a standard sugar-yeast-agar (SYA) medium (100-g brewer’s yeast (*Buy Wholefoods Online*), 50-g sugar (*Tate & Lyle*), 15-g agar, 30-ml Nipagin solution, and 3-ml propionic acid (*Fisher Scientific no. 10193190*) per litre of medium) at a standard density of 100 larvae per vial (i.e. ‘standard density vials’). Experimental flies were then collected under light ice anaesthesia, within 6 h of eclosion to ensure virginity.

#### Development of mathematical model of egg clustering to test for nonrandom egg deposition (H1)

To ascertain whether the proportion of observed egg clustering differed from a null expectation, we produced a bespoke model to calculate random distributions of eggs in the surface area of a standard vial used in all experiments we present here. This model also incorporates the same sized adult female social groups as used in our experiment. Further unique to this model, we defined a ‘cluster’ as any group of two or more eggs in which the main bodies of the eggs were in direct contact (Fig. 1). In the model, given that the observed egg to dish area is 1/5770, we assume that (i) there are 5770 positions available for egg laying, and (ii) there is a total number (parameter Ɛ) of eggs per vial. Assuming that all eggs are laid sequentially and at random, we calculated the expected size of clusters (defined as the number of eggs per cluster) for any number of total eggs laid (Ɛ).

The preference for clustering eggs next to an existing egg (hereafter ‘clustering preference’) is defined by the probability of a clustering decision. *К* is the probability that a fly will lay an egg next to an already existing egg. After the first egg is placed at random, where *Ɛ* > 1, the next egg is either laid in a random position (probability of К — 1) or next to an existing egg (probability of К); in the latter case, the position of the egg is chosen at random among only positions with existing eggs.

The expected number of eggs per cluster can be predicted based on *К* and *Ɛ*. When *К* = 0 (no preference for egg clustering), very few clusters with two eggs occur and virtually no clusters with more than two eggs (2.5% with *Ɛ* = 233 (the maximum number of eggs observed in our experiment), 1% with *Ɛ* = 100, no clusters for *Ɛ* below 70). Therefore, if *Ɛ* < < 5770 (as is the case in our experiment), randomly laid eggs form almost exclusively singly laid eggs. When *К* = 1 (absolute preference for egg clustering), all eggs are clustered in one large cluster in a single position.

At intermediate values of *К*, some variation in cluster size emerges. We simulated the expected number and size of egg clusters for different values of *К* from 0 to 1 in intervals of 0.1 (using the average of 10 different simulations per *К* value). We then compared these expected distributions to the empirically observed distributions of eggs laid by females from the differing social density treatments (groups of one, two, four, or eight) described below to estimate the most likely preference *К* leading to that distribution. It is important to note that these experimental vials were prepared carefully to ensure the laying surface was smooth and contained no depressions to cause any bias in oviposition site choice preferences. For each vial, we performed a Kolmogorov–Smirnov test (implemented in Mathematica 13. 1 [68]) between the expected and observed distribution for all К values. We considered the *К* value yielding the highest (least significant) *P*-values to be the most likely *К* value for that vial.

#### Testing for plasticity in egg-clustering behaviour in response to varying egg and adult density (H2)

##### Effects of adult social density on egg-clustering decisions and fitness

We investigated how variation in adult social group size affected egg-laying and egg-clustering decisions: specifically, the speed of egg laying, the number of eggs laid, egg-clustering patterns, and fitness (egg-adult viability of clustered versus non-clustered eggs). Some data from this experiment were also used to parameterise the model above. Experimental flies were collected and housed in same-sex groups of 10 in standard vials. At 4-day post-eclosion, females were randomly assigned to one of the following social group treatments: one, two, four, or eight females and kept in these conditions for 48 h (*N* = 30 vials each treatment; Additional file 1: Fig. S1). Males were held for 6 days until they were used for matings.

At 6-day post-eclosion, males were introduced to all the female social group size treatment vials for 2 h at a density of 3:2 (males-females) (or 2:1 for isolated females) to enable choice (Additional file 1: Fig. S1). To ensure that this was a set up that would result in all females successfully mating within the 2-h period allowed, we provided additional males to allow females a choice among males and to guard against any individual males being sterile. In addition, we separately recorded female mating frequency in an identical experimental set up. In this, we observed that 97.3% of females mated, with no significant difference in mating frequency between treatments (*χ*^2^ = 1.79, *df* = 3, *p* = 0.617). Thus, we can assume that there is only a very low probability of females remaining unmated by using this procedure.

After the 2-h mating period in the main experiment, we transferred all mated females to fresh standard vials to observe egg-laying decisions. Every 2–3 h, we counted the number of eggs laid, the number of egg clusters, and the size of egg clusters. Observations were made at 14:00, 16:00, 19:00, and 22:00 on day 1 of laying and 10:00 and 12:00 on day 2 (i.e. 2, 4, 7, 10, 22, and 24 h after the end of the mating period, respectively). Differences in counts (number of eggs, number of clusters, and size of clusters) between timepoints were calculated to give overall egg-laying latencies, numbers, and clustering rates. We calculated the proportion of eggs in clusters at each timepoint by summing the total number of eggs in each cluster and dividing by the overall total number of eggs laid in the vial. All females were removed after 12:00 on day 2, and vials were then kept for 14 days to count the total number of eclosed adults from each of the social exposure treatments.

##### Effects of existing egg clusters on female egg‑clustering decisions

To assess whether the size of egg clusters already present in an environment alters subsequent female egg-clustering decisions, we presented focal females with a laying environment that already contained varying numbers of eggs and egg clusters. To create the egg clusters, singly laid eggs were collected within 2 h of laying from SYA medium-filled Petri dishes and transferred into vials to create four different egg cluster sizes: 1, 4, 7, and 10 eggs per cluster. Females have a tendency to lay eggs at vial edges (supplementary information text; Additional file 1: Table S1 [9, 44, 45]); hence, we simulated this preference by placing all egg clusters at the edge of the egg treatment vials.

To obtain the Dahomey females and males for the experiment, we collected virgin flies from standard density vials. Prior to the tests, females were maintained alone and males in groups of four per vial. At 7-day post-eclosion, males and females were paired and observed to ensure they had all successfully mated. After mating, groups of 4 females were then transferred to each of the 4 types of egg cluster treatment vials (1 egg: *N* = 20 vials; 4 eggs: *N* = 17 vials; 7 eggs: *N* = 20 vials; 10 eggs: *N* = 21 vials). After the introduction of the focal females, vials were checked at 30-min intervals to measure rates of oviposition from 13:00 until 21:30, at which point, if females had not laid eggs, they were removed. If eggs were laid by the introduced focal females, we counted the number of eggs laid and categorised them in relation to the existing egg cluster present (clustered or not clustered). All vials were then frozen at − 20 °C for imaging.

Vials were imaged to record inter-egg distances, using a video camera (Sony Handycam HDR-CX405). Using ImageJ’s multiple point selector tool [69], we captured x–y coordinates of each egg and converted these into Euclidean pairwise distances. Selected coordinates were taken from the edge points of eggs that were in closest proximity, resulting in clustered eggs having a distance value of zero.

#### Testing for mixed maternity of egg clusters (H3)

In the final experiment, we determined the extent of egg clusters of single or mixed maternity. In each treatment group, there was one wild type (Dahomey) focal and three non-focal females from the *scarlet* strain (recessive *scarlet* eye colour mutation backcrossed into the Dahomey wild type > 4 times). These non-focal *scarlet* females were fed Sudan Black B dye that stained laid eggs, allowing the eggs of focal versus non-focal females to be identified. This combination of strain and dye-feeding enabled us to identify eggs and adult flies. First instar larvae of Dahomey wild type and *scarlet* strains were raised in cultures of 100 larvae per vial as described above. Focal Dahomey females were collected from cultures reared and maintained on standard medium, whereas *scarlet* females were derived from cultures containing Sudan Black B dye (100-g brewer’s yeast, 50-g sugar, 15-g agar, 30-ml Nipagin solution, 3-ml propionic acid, and 1.4-g Sudan Black B powder (*Sigma-Aldrich,* cat. no. 199664) dissolved in 14-ml corn oil (*Mazola* 100% pure corn oil) per litre of medium). Focal Dahomey virgin females were collected from the standard medium vials, and non-focal *scarlet* virgin females were collected from Sudan Black B vials and maintained on that same medium until use. Females were initially housed in groups of 10; then, at 5 days post-eclosion, they were randomly assigned to groups of 1 Dahomey female and 3 *scarlet* females per standard SYA vial. In parallel, a second set of *scarlet* females were housed in groups of four in Sudan Black B vials. Therefore, there were two sets of *scarlet* females — those used in the pre-mating social environment and those used in the oviposition assay. This was necessary to maintain the focal Dahomey female on standard undyed food (but still expose the focal fly to *scarlet* conspecifics before mating) and the ovipositing *scarlet* females on dyed food to maintain the intensity of the dye. The s*carlet* females that were co-housed with Dahomey females before mating were wing clipped for ease and speed of identification without the use of a microscope. Seven days post-eclosion, males were transferred into the female vials for mating: eight Dahomey males were added to each group of one Dahomey: three *scarlet* females and six *scarlet* males for each group of four *scarlet* females. The flies were given 4 h to mate. The Dahomey female was then transferred to fresh standard media alongside three randomly chosen *scarlet* dye-fed females at 12:00 GMT (*N* = 29) (the three wing-clipped scarlet females that had been co-housed with the focal female before mating were discarded). We then counted the number of eggs laid at 10.5-h post-mating. Eggs were categorised as clustered or not clustered (Fig. 1) and according to whether they were laid by a focal or non-focal female. This allowed us to calculate the proportion of eggs that were clustered and whether the cluster contained eggs from both focal and non-focal females.

### Statistical analysis

All statistical analyses were conducted using R v 4.2.2 [70], and mean values and standard deviations are reported within the text. Graphs were produced using packages ‘ggplot2’, ‘cowplot’, and ‘grid’ [71, 72], with plotted standard errors calculated using ‘Rmisc’ [73].

We analysed the effect of egg and adult social density on the likelihood that females laid eggs and produced adult offspring by using generalised linear models (GLMs) with binomial errors. We used the package ‘AER’ [74] to test for overdispersion, and where that was found, we used GLMs with a quasi-Poisson distribution to account for this overdispersion and analyse the effect of adult density on the number of offspring produced. Linear models were used to investigate the effect of adult density on egg-clustering patterns (egg-laying and-clustering rates), and total eggs laid was included as a fixed effect to account for increased likelihood of clustering when more eggs are present in the environment.

When analysing variables throughout multiple timepoints, we used linear mixed-effects models; the timepoint was included as a fixed effect in the model to account for differences in rates, and the test vial was included as a random effect — using functions in packages ‘lme4’ [75] and ‘lmerTest’ [76]. We tested for Pearson’s product-moment correlations between the proportion of eggs laid in clusters and subsequent egg-adult viability. In all cases, post hoc tests (models including only pairwise comparisons) were used to identify significant differences in the six pairwise comparisons available.

In the first experiment investigating the effects of social density, there was variation in sampling effort between treatments, due to the differences in female group sizes (and lack of identification of focal females). To account for this, all significant results from the initial analyses were reanalysed with a randomised subset of data so that the number of individual flies (rather than number of vials) per treatment was similar. In this reduced subset, all vials from solitary females were included (*N* = 30 females), half of those in pairs (*N* = 30 females), eight of those from groups of four (*N* = 32 females), and four of those from groups of eight (*N* = 32 females). These analyses were congruent with those of the initial analyses, suggesting the initial analyses were robust.

The datasets generated and/or analysed during the current study are openly available from the Environmental Information Data Centre [77].

## Supplementary Information

Additional file 1: Supplementary text 1. Determination of effect of natural egg placement and egg ‘edge effects’ on subsequent egg laying behaviour. Figure S1. Experimental set up for testing the effects of social densities onegg laying. Figure S2. Existing egg location did not impact latency to lay. Figure S3. Females in larger social groups laid significantly larger clusters. Figure S4. Females in larger social groups laid significantly more clusters. Figure S5. No evidence of fitness effects of social environment. Table S1. Edge effect egg laying location decisions were not overridden by adult social group size or existing egg locations. Table S2. Egg laying patterns of females were significantly non-random.

## Data Availability

The datasets generated and/or analysed during the current study are openly available from the Environmental Information Data Centre [77] (10.5285/0854f2de-137f-472f-9c0c-9ae1f69e7f19).

## Abbreviations

H: Hypothesis
K: Clustering preference (described in the model)
Ɛ: Total theoretical eggs (described in the model)
LM: Linear model
GLM: Generalised linear model
LME: Linear mixed-effects model
SYA: Standard sugar-yeast-agar medium

## Declarations

### Authors’ social media handles

Bluesky:

Emily R. Churchill: @emilyrosech.bsky.social.‬

Amanda Bretman: @amandabretman.bsky.social.‬
