# Supplementary material for: Female fruit flies use social cues to make egg-clustering decisions
Source: BMC Biol. 2025 Oct 14;23:306. doi: 10.1186/s12915-025-02382-w (PMC12522869; doi:10.1186/s12915-025-02382-w)
Supplement: Supplementary file 1 — Additional file 1: Supplementary text 1. Determination of effect of natural egg placement and egg ‘edge effects’ on subsequent egg laying behaviour. Figure S1. Experimental set up for testing the effects of social densities onegg laying. Figure S2. Existing egg location did not impact latency to lay. Figure S3. Females in larger social groups laid significantly larger clusters. Figure S4. Females in larger social groups laid significantly more clusters. Figure S5. No evidence of fitness effects of social environment. Table S1. Edge effect egg laying location decisions were not overridden by adult social group size or existing egg locations. Table S2. Egg laying patterns of females were significantly non-random. [file 12915_2025_2382_MOESM1_ESM.docx]

**Supplementary Information**

***Supplementary text 1. Determination of effect of natural egg placement and egg ‘edge effects’ on subsequent egg laying behaviour***

In this study, we tested the responses of females to the presence of existing eggs of various cluster sizes in the environment. This required us to manually place eggs in vials to create the required variation in egg clustering. Previous research shows that females naturally prefer to lay eggs at the edges of substrates (9, 47, 48). Such edge biases could themselves influence the decisions of subsequent females about whether to create egg clusters. To test for this, and thus understand how best to place eggs in the main experiment, we conducted an initial preliminary experiment to test whether proximity of existing eggs to the edge off the vial affected the subsequent latency of females to lay eggs near them.

We tested isolated versus grouped females laying eggs in 3 types of ‘egg treatment’ vials: in which there was (i) no egg present (control), and (ii) 1 egg either placed in the centre or (iii) at the edge of the vial. The non-focal eggs used to create the egg treatment vials were laid by groups of 10 gravid females allowed to lay overnight on Petri dishes filled with 40ml of SYA medium. To standardise eggs, we selected only eggs laid naturally in isolation from these dishes for subsequent transfer to create the egg treatment vials. To enable transfer of eggs without damage to the SYA medium, elected eggs were transferred using a mounted needle, into standard plastic vials that had been cut in half. Then the top half was reattached after egg transfer using Sellotape. Test flies were collected from standard density vials (as in the main experiments) and unmated females were housed in solitude, and males in groups of four. After six days, one male was transferred into the vial with each female, and pairs were watched to ensure matings occurred. We then transferred females in isolation or in groups of 4 into vials containing the 3 egg treatments (no egg, 1 edge egg, or 1 central egg; N = 15 - 42 vials per treatment).

We then checked vials every 30 minutes until 21:30 (one count after lights off in the constant environment room) and removed the female(s) if they had laid eggs. If they did not lay, they were kept overnight, and egg counts resumed at 09:30 for three further hours, until 24 hours after females had initially been introduced into the vials. We calculated egg laying latencies and the number of eggs laid by the focal females. We also noted whether eggs were laid in a cluster (defined in Fig. 1) alongside the existing egg.

The results revealed a strong preference to lay at the edge of the vial, confirming previous reports. There was no effect of existing egg location (X^2^ = 1.32, d.f. = 113, p = 0.517) or group size (X^2^ = 3.80, d.f. = 112, p = 0.0513) on where in the environment females subsequently laid their eggs. There was also no effect of either egg location or group size on whether females chose to cluster their eggs as no females clustered their eggs (Table S1). Females housed in solitude took longer to initiate laying than did those in groups of four (X^2^ = 4.38, d.f. = 126, p = 0.001; Fig. S2). There was no effect of existing egg location on laying latency (X^2^ = 1.30, d.f. = 125, p = 0.139; Fig. S2). Based on these results, we created egg clusters in the main experiments by following the natural patterns by placing eggs at the vial edges.

***Figure S1. Experimental set up for testing the effects of social densities on egg laying***


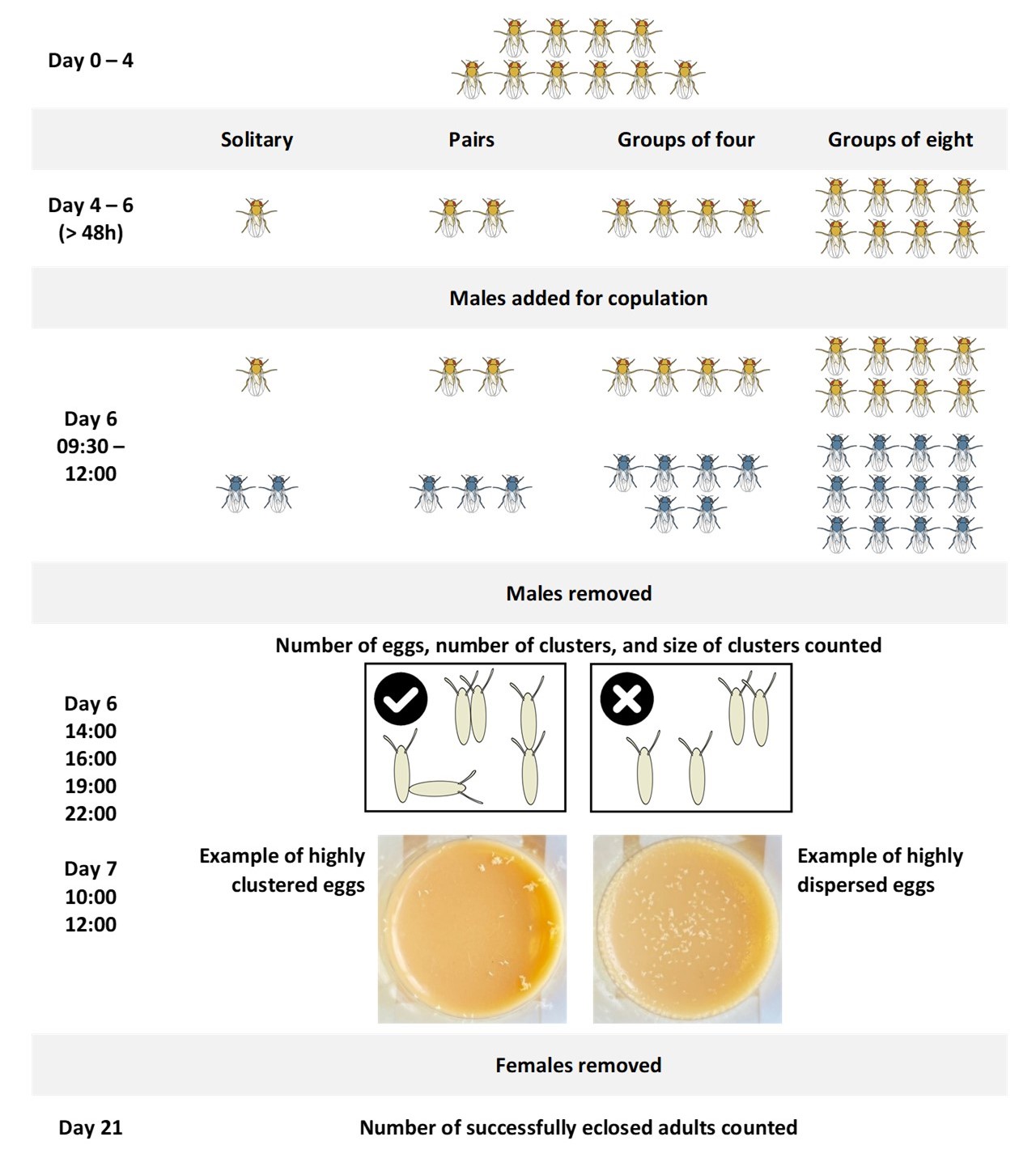


Figure S1. Summary of the experimental set up for testing the effects of social densities on egg laying. Day 0 – 4: males and females housed in same-sex groups of 10. Day 5 – 6: females transferred to one of four social treatments (whilst males remained in groups of 10). Day 6: males were added to female treatment vials in a ratio of 3:2 (2:1 for isolated females) and given 2h to mate. Day 6 – 7: number of eggs, number of clusters and size of clusters were counted at the six listed timepoints. Shown is the strategy for defining egg clusters (contact between the main body of eggs).

***Figure S2. Existing egg location did not impact latency to lay***


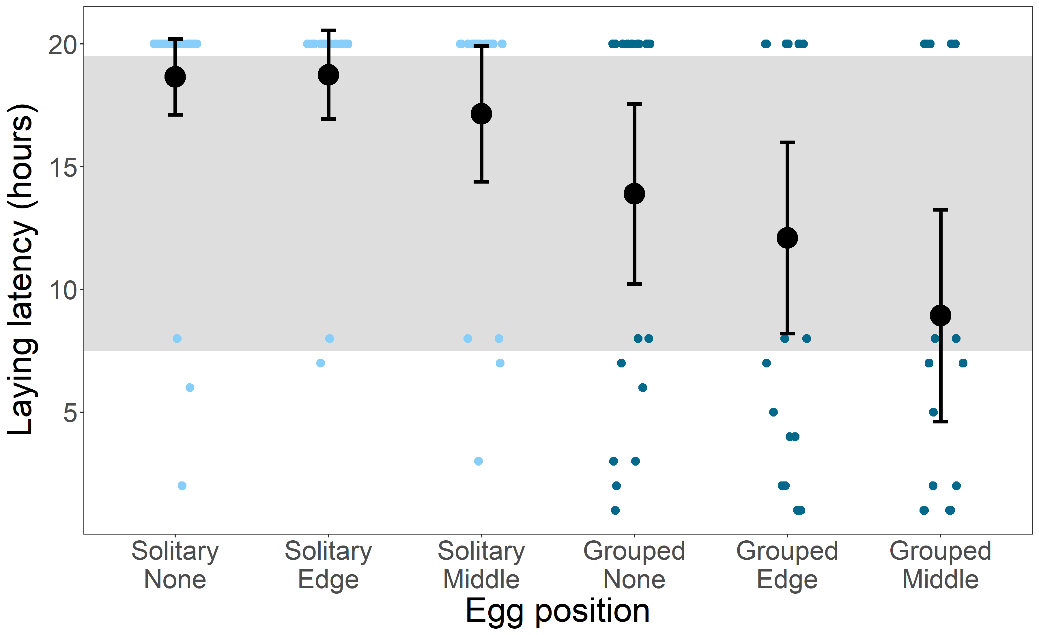


Figure S2. Females housed in solitude were slower to start laying than those housed in groups of 4, but there was no effect of existing egg location on laying latency. Means and standard errors are shown for the six treatments. Grey box indicates period of dark (21:00 – 09:00 GMT). N = 30 vials for all four social treatments.

***Figure S3. Females in larger social groups laid significantly larger clusters***


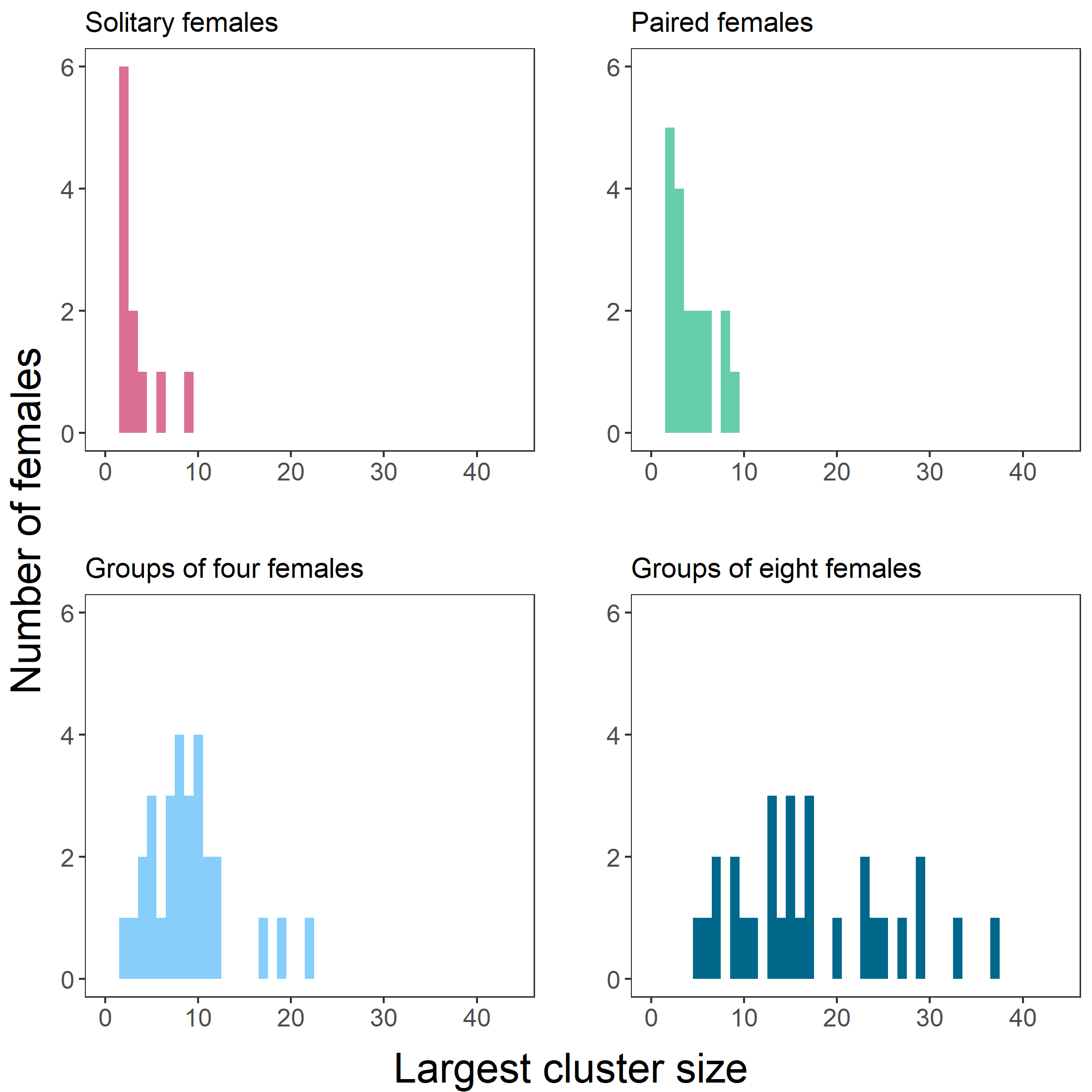


Figure S3. Maximum cluster size increased with increasing female social density. Shown is a frequency distribution of the largest egg cluster for mated females placed in social groups of 1, 2, 4, and 8 for 24 hours. Clusters were defined as in Fig. 1. Data are shown as the total number of vials with each cluster size observed for each of the four treatments (solitary females: N = 11; pairs: N = 18; groups of 4: N = 29; groups of 8: N = 30 vials).

***Figure S4. Females in larger social groups laid significantly more clusters***


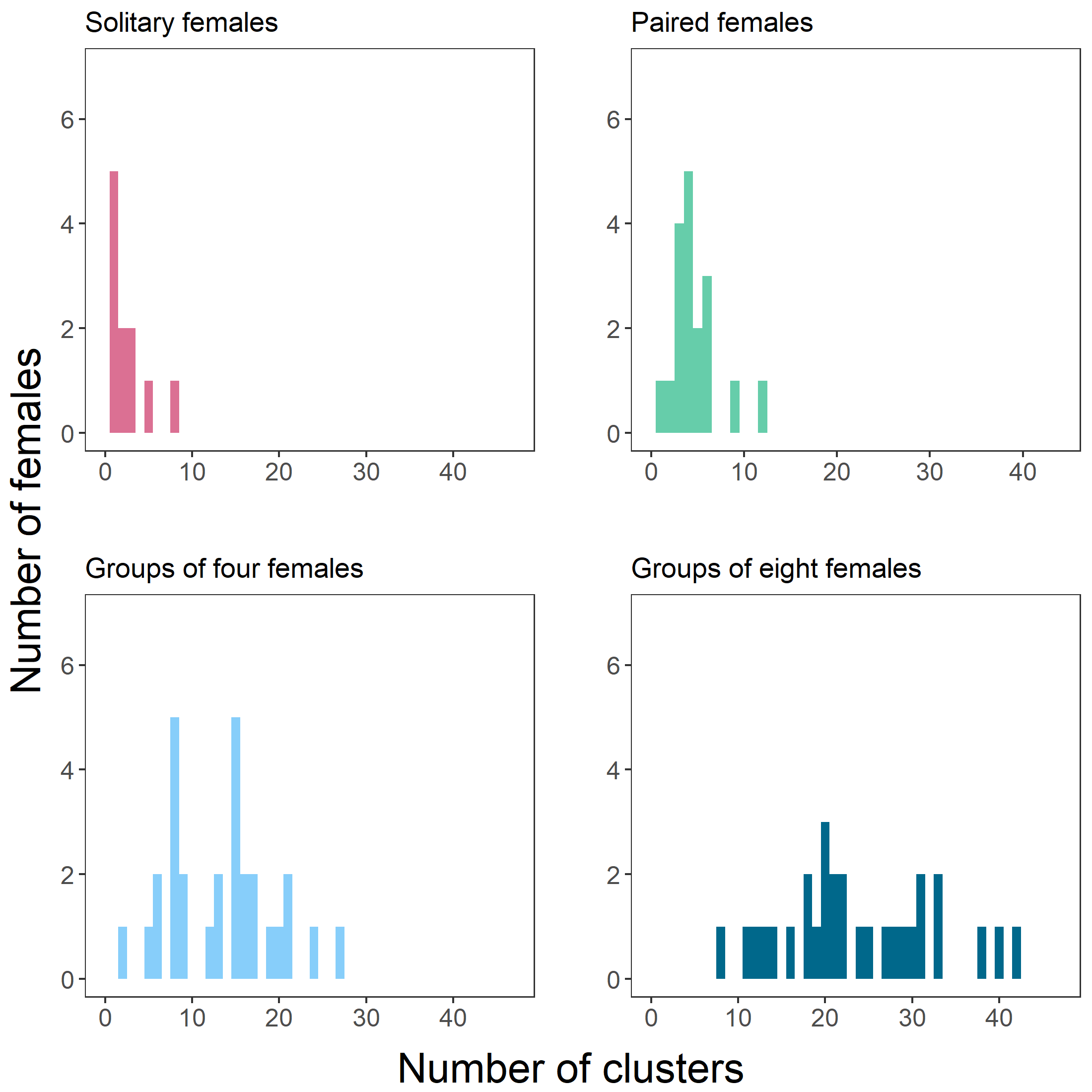


Figure S4. A greater number of clusters were observed with increasing female social density. Shown is a frequency distribution of the egg clustering sizes for mated females placed in social groups of 1, 2, 4, and 8 for 24 hours. Clusters were defined as in Fig. 1. Data are shown as the total number of vials with each cluster size observed for each of the four treatments (solitary females: N = 11; pairs: N = 18; groups of 4: N = 29; groups of 8: N = 30 vials).

***Figure S5. No evidence of fitness effects of social environment***


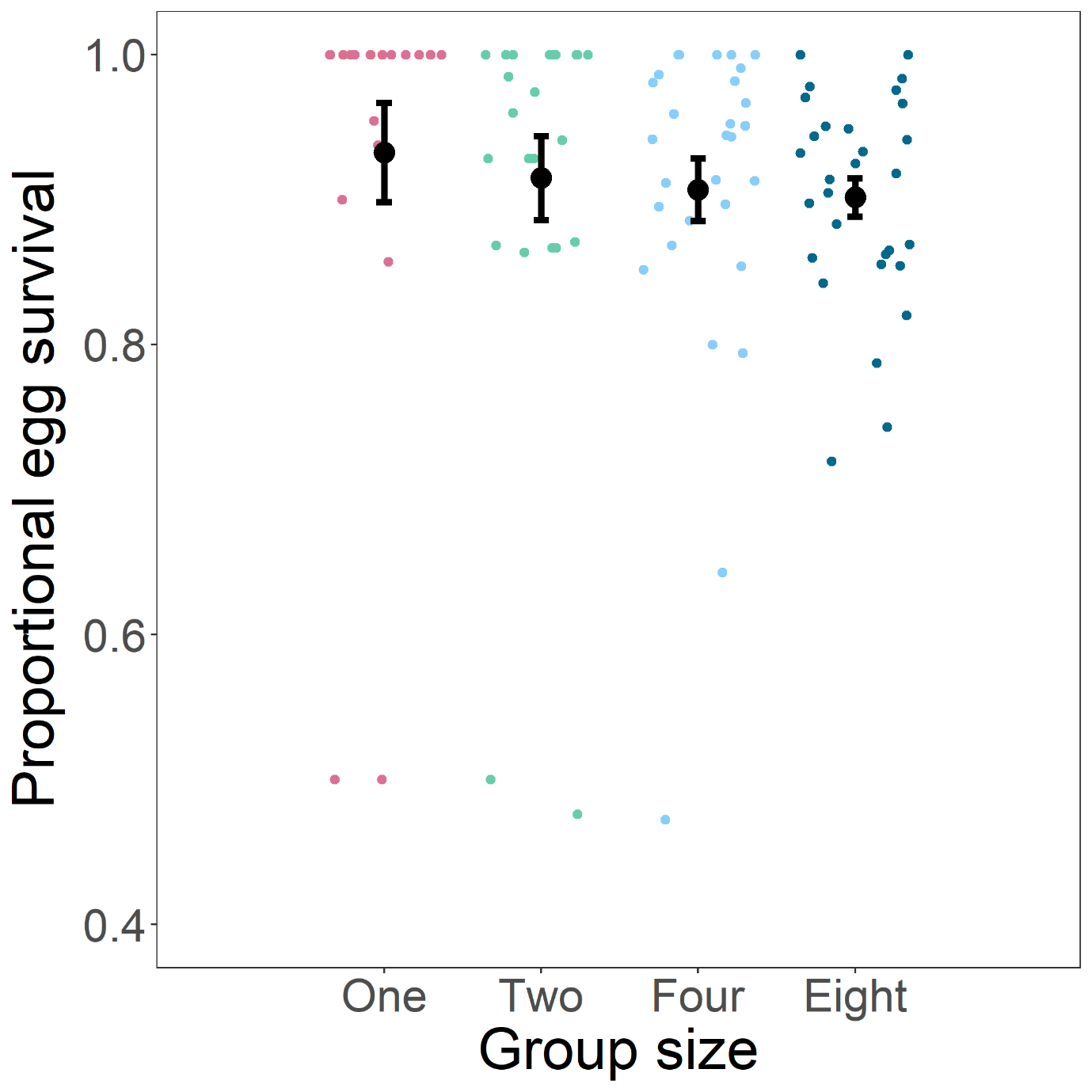


Figure S5. There was no effect of social group size on egg-adult viability (solitary: N = 20 vials; paired: N = 24 vials; groups of 4: N = 29 vials; groups of 8: N = 30 vials). Means (large dot) and standard errors are shown in black.

***Table S1. Edge effect egg laying location decisions were not overridden by adult social group size or existing egg locations***

Table S1. Effect of social density and previous egg laying patterns on female fecundity and egg clustering. The proportion of vials where eggs laid were observed to be clustered with the existing egg (main bodies in direct contact) and laid at the edge of the vial (within 0.5cm from the vial circumference).

|  |  | Proportion of eggs laid at the edge (%) | Proportion of eggs clustered (%) |
| --- | --- | --- | --- |
| Social treatment | Egg location |  |  |
| Solitary females | None (N = 33 vials) | 100 | - |
|  | Edge (N = 22 vials) | 100 | 0 |
|  | Centre (N = 15 vials) | 100 | 0 |
| Groups of four | None (N = 17 vials) | 94.1 | - |
|  | Edge (N = 17 vials) | 93.8 | 0 |
|  | Centre (N = 13 vials) | 100 | 0 |

***Table S2. Egg laying patterns of females were significantly non-random***

Table S2. Comparison of the clustering proportion model outputs and empirical egg laying patterns observed in *D. melanogaster*. Shown are Kolmogorov-Smirnov P value outputs for the comparison of empirical data to expected clustering preference values (К = 0 to К = 1, in 0.1 increments) rounded to two decimal places. Values that show the most likely clustering preference are highlighted in bold.

| **Social treatment** | **К values** | | | | | | | | | | |
| --- | --- | --- | --- | --- | --- | --- | --- | --- | --- | --- | --- |
|  | 0.0 | 0.1 | 0.2 | 0.3 | 0.4 | 0.5 | 0.6 | 0.7 | 0.8 | 0.9 | 1.0 |
| Solitary  N = 20 vials | 0.09 | 0.34 | 0.44 | **0.48** | 0.46 | 0.29 | 0.17 | 0.06 | 0.02 | 0.03 | 0.01 |
| Paired  N = 25 vials | 0.01 | 0.20 | 0.34 | 0.43 | **0.43** | 0.38 | 0.22 | 0.15 | 0.07 | 0.04 | 0.01 |
| Groups of four  N = 29 vials | 0.00 | 0.08 | 0.16 | 0.25 | **0.29** | 0.29 | 0.23 | 0.13 | 0.05 | 0.02 | 0.00 |
| Groups of eight  N = 30 vials | 0.00 | 0.00 | 0.07 | 0.15 | 0.20 | **0.21** | 0.15 | 0.08 | 0.03 | 0.01 | 0.00 |
